# Novel Risk Variants in the Oxytocin Receptor Gene (*OXTR*) Possibly Linked to and Associated with Familial Type 2 Diabetes

**DOI:** 10.3390/ijms24076282

**Published:** 2023-03-27

**Authors:** Mutaz Amin, Rongling Wu, Claudia Gragnoli

**Affiliations:** 1 French National Institute of Health and Medical Research (INSERM), US14-Orphanet, 75014 Paris, France; 2 Department of Biochemistry and Molecular Biology, Faculty of Medicine, Al-Neelain University, Khartoum 11121, Sudan; 3 Department of Public Health Sciences, Penn State College of Medicine, Hershey, PA 17033, USA; 4 Department of Statistics, Penn State College of Medicine, Hershey, PA 17033, USA; 5 Division of Endocrinology, Department of Medicine, Creighton University School of Medicine, Omaha, NE 68124, USA; 6 Molecular Biology Laboratory, Bios Biotech Multi-Diagnostic Health Center, 00197 Rome, Italy

**Keywords:** oxytocin, OXT, oxytocin receptor, *OXTR*, type 2 diabetes, T2D, single nucleotide polymorphisms, SNPs, linkage, linkage disequilibrium, association

## Abstract

The oxytocin system is well-known for its role in social bonding and reproduction. Recently, the oxytocin system was found to play other metabolic roles such as regulation of food intake, peripheral glucose uptake, and insulin sensitivity. Variants in *OXTR* gene have been associated with overeating, increased cardiovascular risk, and type 2 diabetes (T2D). We tested 20 microarray-derived single nucleotide polymorphisms in the *OXTR* gene in 212 Italian families with rich family history for T2D and found four novel and one previously reported variant suggestively significant for linkage and association with the risk of T2D. Our study has shed some light into the genetics of susceptibility to T2D at least in Italian families.

## 1. Background

Oxytocin (OXT) is a neuropeptide hormone produced by the hypothalamus and secreted by the posterior pituitary gland [1]. Oxytocin notably plays important roles in social bonding and reproduction [2,3]. Recent evidence suggests that oxytocin also plays other metabolic roles such as regulation of food intake [4], peripheral glucose uptake [5], insulin sensitivity [6], β-cell function and survival, and insulin secretion [7], which prompted investigating the implication of oxytocin pathway in type 2 diabetes (T2D). Of note, administration of oxytocin in humans improved insulin sensitivity and responsiveness of pancreatic β-cells [8]. The oxytocin receptor (OXTR) is a G-protein coupled receptor that mediates the central and peripheral actions of oxytocin [9]. It is encoded by the oxytocin receptor gene (*OXTR*) and is expressed in a variety of central and peripheral tissues such as the brain, heart, kidneys and pancreas [10]. *OXTR*-/- knockout mice show insulin insensitivity and impaired glucose tolerance and lack protection from cell death induced by cytotoxic and metabolic stress [11], and variants in *OXTR* gene have been associated in humans with overeating [12], increased cardiovascular risk [13] and T2D [14], and they possibly act through regulation of glucose tolerance and insulin sensitivity [15,16] The association of *OXTR*-risk variants with T2D has been found in population-based case control studies [14], but familial studies which are powerful in detecting linkage signals, not common and rare variants, and inheritance models underlying the conferred risk, are still lacking. In this study, we aimed at analyzing *OXTR* gene variants for familial linkage with T2D.

## 2. Results and Discussion

**Figure 1:**
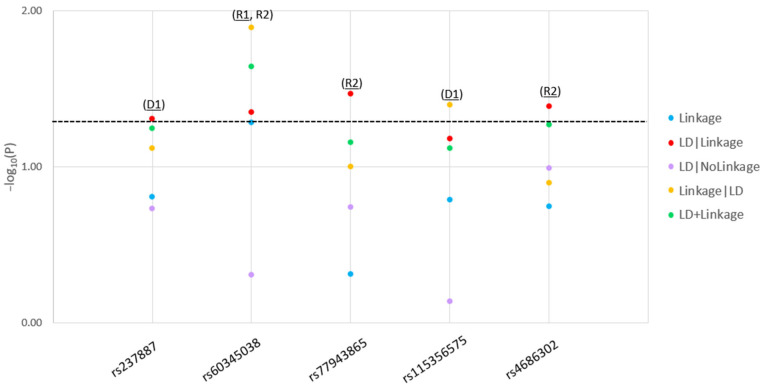
T2D *OXTR* risk single nucleotide polymorphisms (SNPs) linkage and linkage-disequilibrium (LD) analysis results. **Legend:** For each significant risk single nucleotide polymorphism (SNP) in the *OXTR* gene, we present the −log_10_(P) as a function of each test statistic (Linkage, linkage disequilibrium [LD]|Linkage, LD|Nolinkage, Linkage|LD, and LD+Linkage) and label the inheritance model: D1: dominant, complete penetrance, R1: recessive, complete penetrance, R2: recessive, incomplete penetrance. For each SNP we present the most significant test statistics across the significant models.

Five variants (rs237887, rs60345038, rs77943865, rs115356575 and rs4686302) were suggestively significant for linkage to and linkage disequilibrium (LD) with T2D (*p* < 0.05) (Figure 1) across different inheritance models (Table 1). All variants were independent (i.e., no LD blocks were found). With the exception of (rs237887), all variants are novel and have not been reported before with T2D or other related phenotype. The non-risk allele (G) of the variant (rs237887) was part of a haplotype reported to be weakly associated with the protection against T2D in a case-control study [14]. We performed in silico functional predictions for the suggestively significant single nucleotide polymorphisms (SNPs) in our study (pathogenicity [SIFT] [17], splicing [SpliceAI] [18], transcription-factor binding [SNPnexus] [19] and SNP2TFBS [20], regulatory potential [RegulomeDB] [21], and miRNA binding [mirSNP] [22]) and predicted that all T2D risk variants were associated with repressed chromatin state in the endocrine pancreas which potentially leads to negative expression of the *OXTR* gene [21]. We found no functional predictions related to affected binding of transcription factors, miRNAs or impaired splicing. These findings however, are consistent with the T2D-related phenotypes (decreased insulin sensitivity and impaired glucose tolerance) observed in *OXTR*-/- knockout mice [11]. The results of ours study indicate that *OXTR*-variants may confer risk not only to sporadic cases [14] but also to familial T2D, at least in Italian families. The *OXTR* gene is expressed in both the brain and the endocrine pancreas [10]; its implication in T2D could be mediated by its role in appetite [12] and/or insulin sensitivity [15,16]. However, the variants in our study are only suggestively significant since correction for multiple comparison testing has not been applied. Therefore, functional and replication studies in diverse ethnic groups are still needed to validate these results.

## 3. Methods

### 3.1. Patients

We recruited 1156 subjects from 212 Italian families with rich family history of T2D diagnosed following the National Diabetes Data Group Criteria (22): “Presence of the classical signs and/or symptoms of diabetes plus elevated hyperglycemia, or by elevated fasting plasma glucose ≥140 mg/dL”. This was followed by reviewing the American Diabetes Association criteria: fasting glycemia at 126 mg/dL or higher in at least two measurements and/or random glycemia of at least 200 mg/dL or higher with symptoms, and/or at least 200 mg/dL or higher 2 h after an oral glucose tolerance test of 75 mg of glucose, after excluding secondary causes of diabetes (e.g., pancreatectomy). According to these criteria, 650 patients were diagnosed with T2D. The average male:female ratio was 1.04. The age of onset of T2D ranged from 7 to 81 years with a mean age of 47.8.

### 3.2. Genetic Testing and Analysis

We tested 20 microarray-derived SNPs in the *OXTR* gene (Supplementary Table S1). The SNPs were amplified using Affymetrix SNP array. PLINK tool was used to exclude Mendelian and genotyping errors [23]. Using Pseudomarker [24], we performed 2-point parametric linkage and linkage-disequilibrium (LD) analyses with T2D, initially testing the recessive model with incomplete penetrance (R2). Subsequentially, we ran a secondary analysis with the models of recessive complete penetrance (R1), dominant complete penetrance (D1) and dominant incomplete penetrance (D2). Each SNP was tested for 1. Linkage, 2. LD|Linkage, 3. LD|NoLinkage, 4. Linkage|LD and 5. LD+Linkage. We report in Supplementary Table S1 the *p* value of the following analyses: LD/linkage, the most important, as it tests for LD given the presence of linkage; linkage/LD, which tests for linkage given the presence of LD; and LD + linkage tests, which is a combinatorial test. Presence or absence of LD blocks was imputed according to LD correlations from SNPs available in the Tuscany Italian population from the 1000 Genomes Project (https://www.internationalgenome.org/data-portal/population/TSI [accessed on 15 September 2022]). SNPs with r^2^ ≥ 0.9 were considered “in LD” (Supplementary Table S2). The study was approved by the Bios Ethical Committee.

## Figures and Tables

**Table 1:** *OXTR* risk single nucleotide polymorphisms (SNPs) linked/in linkage-disequilibrium (LD) to/with type 2 diabetes (T2D).

Model ^1^	SNP	Position	Ref	Alt	Risk Allele	Consequence	RAF ^2^	P ^3^	Reported in T2D?
D1	rs237887	8755356	G	A	A	Intronic	0.54	0.04	Yes [14]
R1, R2	rs60345038	8760830	C	T	C	Intronic	0.62	0.01	Novel
R2	rs77943865	8762293	G	A	G	Intronic	0.95	0.03	Novel
D1	rs115356575	8764840	G	A	G	Intronic	0.96	0.04	Novel
R2	rs4686302	8767536	C	T	C	Missense	0.89	0.04	Novel

**Legend.** ^1^ Models: D1: dominant, complete penetrance, R1: recessive, complete penetrance, R2: recessive, incomplete penetrance. ^2^ RAF: risk allele frequency. ^3^ P: *p*-value of the most significant test statistics. The most significant model is underlined.

## Data Availability

The study data are available on reasonable request; due to lacking specific patients’ consent and privacy restrictions, they are not publicly available.

## Supplementary Materials

The following supporting information can be downloaded at: https://www.mdpi.com/article/10.3390/ijms24076282/s1.
