# Supplementary material for: Novel Risk Variants in the Oxytocin Receptor Gene (OXTR) Possibly Linked to and Associated with Familial Type 2 Diabetes
Source: Int J Mol Sci. 2023 Mar 27;24(7):6282. doi: 10.3390/ijms24076282 (PMC10094736; doi:10.3390/ijms24076282)
Supplement: Supplementary file 1 [file ijms-24-06282-s001.zip › ijms-1946263-supplementary.pdf]

Table S1. OXTR-SNPs tested in Italian families with T2D. List of the 20 SNPs located within the OXTR gene, including the P value of the following analyses: LD/linkage, linkage/LD, and LD + linkage.

Marker	Model	LD|Linkage	Linkage|LD	LD+Linkage
rs11131147	D1	0.145799	0.38108	0.246514
rs1042778	D1	0.932861	1	0.964694
rs11706648	D1	0.43457	0.475067	0.412904
rs237887	D1	0.0491	0.076305	0.056625
rs918316	D1	0.253764	1	0.387598
rs116571980	D1	0.50711	1	0.654799
rs115396476	D1	0.802052	1	0.862448
rs2254295	D1	0.886749	0.697847	0.802068
rs60345038	D1	0.400535	0.081377	0.124115
rs237891	D1	0.847529	1	0.914609
rs77943865	D1	0.114009	0.116407	0.200418
rs35498753	D1	0.057559	0.501747	0.104749
rs115356575	D1	0.066184	0.040051	0.075848
rs2268495	D1	0.624099	0.190433	0.237582
rs61183828	D1	0.155677	0.254593	0.260351
rs237900	D1	0.735687	1	0.84015
rs61740241	D1	0.282111	0.350106	0.421452
rs4686302	D1	0.139914	0.22337	0.14743
rs2228485	D1	0.115528	0.080183	0.133542
rs62243375	D1	0.304044	1	0.430423

Marker	Model	LD|Linkage	Linkage|LD	LD+Linkage
rs11131147	D2	0.083667	0.510963	0.153829
rs1042778	D2	0.747826	1	0.845662
rs11706648	D2	0.200198	0.576166	0.280702
rs237887	D2	0.136393	0.170351	0.19598
rs918316	D2	0.914327	1	0.95428
rs116571980	D2	0.503016	1	0.651043
rs115396476	D2	0.924297	0.899689	0.942611
rs2254295	D2	0.532906	1	0.678098
rs60345038	D2	0.174773	0.224822	0.175445
rs237891	D2	0.737707	1	0.841585
rs77943865	D2	0.328882	0.581437	0.474872
rs35498753	D2	0.72721	0.843484	0.834054
rs115356575	D2	0.062995	0.107102	0.093294
rs2268495	D2	0.774376	0.814072	0.651374
rs61183828	D2	0.746874	1	0.848056
rs237900	D2	0.971622	1	0.985498
rs61740241	D2	0.321255	0.44651	0.466362
rs4686302	D2	0.113045	0.998871	0.181853
rs2228485	D2	0.065688	0.8721	0.124389
rs62243375	D2	0.167081	0.679308	0.216466

Marker	MODEL	LD|Linkage	Linkage|LD	LD+Linkage
rs11131147	R1	0.218171	0.775892	0.34336
rs1042778	R1	0.637658	0.927441	0.766333
rs11706648	R1	0.45891	0.600649	0.551688
rs237887	R1	0.125318	0.188853	0.111305
rs918316	R1	0.469278	0.446877	0.522083
rs116571980	R1	0.522125	1	0.668441
rs115396476	R1	0.872738	1	0.799662
rs2254295	R1	0.474313	0.706985	0.615771
rs60345038	R1	0.044572	0.012759	0.022651
rs237891	R1	0.577272	1	0.640072
rs77943865	R1	0.158753	0.300857	0.264616
rs35498753	R1	0.14818	0.387575	0.112352
rs115356575	R1	0.440252	0.311886	0.449517
rs2268495	R1	0.706401	0.530967	0.41607
rs61183828	R1	0.093047	0.413529	0.168543
rs237900	R1	0.267886	0.407501	0.391166
rs61740241	R1	0.117475	0.224625	0.205574
rs4686302	R1	0.148908	0.294875	0.192067
rs2228485	R1	0.21026	0.27257	0.217457
rs62243375	R1	0.466988	1	0.617265

Marker	MODEL	LD|Linkage	Linkage|LD	LD+Linkage
rs11131147	R2	0.114287	0.705706	0.200838
rs1042778	R2	0.574997	0.901771	0.714759
rs11706648	R2	0.162327	0.407663	0.230609
rs237887	R2	0.189171	0.197062	0.144209
rs918316	R2	0.779773	0.73785	0.750567
rs116571980	R2	0.414442	1	0.565601
rs115396476	R2	0.442784	0.457941	0.593829
rs2254295	R2	0.672547	0.698116	0.751596
rs60345038	R2	0.027526	0.01511	0.014908
rs237891	R2	0.543422	1	0.546132
rs77943865	R2	0.034101	0.09953	0.069956
rs35498753	R2	0.986376	0.200319	0.252676
rs115356575	R2	0.138072	0.119222	0.150265
rs2268495	R2	0.658632	0.263429	0.240676
rs61183828	R2	0.383327	0.693693	0.533595
rs237900	R2	0.571835	0.514318	0.633534
rs61740241	R2	0.070723	0.144918	0.133015
rs4686302	R2	0.040967	0.12619	0.053336
rs2228485	R2	0.733033	0.312017	0.43572
rs62243375	R2	0.448943	1	0.599778

Table S2. Results of LD matrix (correlation coefficient) of OXTR-tested SNPs in T2D. SNPs with r2 ≥ 0.9 were considered “in LD”, otherwise were considered independent. Our SNPs are independent.

rs_number	rs11131147	rs1042778	rs11706648	rs237887	rs918316	rs202112459	rs116571980	rs115396476	rs2254295	rs60345038	rs237891	rs77943865	rs35498753	rs115356575	rs2268495	rs61183828	rs237900	rs61740241	rs4686302	rs2228485	rs62243375
**rs11131147**	1	0.094	0.022	0.004	0.009	NA	0.045	0	0.003	0.062	0.01	0.008	0	0.002	0.036	0	0.017	0.003	0	0.008	0.001
**rs1042778**	0.094	1	0.091	0.271	0.032	NA	0.074	0.019	0.05	0.166	0.013	0.019	0	0.026	0.007	0.003	0.034	0.002	0.001	0	0.048
**rs11706648**	0.022	0.091	1	0.4	0.026	NA	0.033	0.014	0.051	0.158	0.04	0.004	0.003	0.035	0.012	0	0.005	0	0.051	0.012	0.047
**rs237887**	0.004	0.271	0.4	1	0.063	NA	0.079	0.034	0.157	0.295	0.013	0.033	0.005	0.014	0.074	0	0.015	0.019	0.075	0.002	0.035
**rs918316**	0.009	0.032	0.026	0.063	1	NA	0.005	0.002	0.013	0.031	0.019	0.03	0	0.001	0	0.002	0.003	0.001	0.002	0.02	0.002
**rs202112459**	NA	NA	NA	NA	NA	NA	NA	NA	NA	NA	NA	NA	NA	NA	NA	NA	NA	NA	NA	NA	NA
**rs116571980**	0.045	0.074	0.033	0.079	0.005	NA	1	0.003	0.016	0.17	0.004	0.121	0.001	0.001	0.001	0.001	0.034	0.126	0.006	0.025	0.009
**rs115396476**	0	0.019	0.014	0.034	0.002	NA	0.003	1	0.007	0.016	0.003	0.001	0.006	0	0.009	0.001	0.011	0.001	0.004	0.01	0.004
**rs2254295**	0.003	0.05	0.051	0.157	0.013	NA	0.016	0.007	1	0.093	0.001	0.007	0.183	0.003	0.005	0.001	0.041	0.004	0.012	0.104	0.012
**rs60345038**	0.062	0.166	0.158	0.295	0.031	NA	0.17	0.016	0.093	1	0.1	0.024	0	0.03	0.085	0.002	0.005	0.016	0.068	0.044	0.028
**rs237891**	0.01	0.013	0.04	0.013	0.019	NA	0.004	0.003	0.001	0.1	1	0	0	0.019	0.047	0.022	0.001	0.002	0.003	0.016	0.011
**rs77943865**	0.008	0.019	0.004	0.033	0.03	NA	0.121	0.001	0.007	0.024	0	1	0.138	0	0.131	0.009	0.026	0.001	0.209	0.01	0.004
**rs35498753**	0	0	0.003	0.005	0	NA	0.001	0.006	0.183	0	0	0.138	1	0.002	0	0.007	0.082	0.003	0.028	0.244	0.019
**rs115356575**	0.002	0.026	0.035	0.014	0.001	NA	0.001	0	0.003	0.03	0.019	0	0.002	1	0.004	0.001	0.019	0	0.002	0.004	0.002
**rs2268495**	0.036	0.007	0.012	0.074	0	NA	0.001	0.009	0.005	0.085	0.047	0.131	0	0.004	1	0.17	0.195	0.005	0.443	0.209	0.03
**rs61183828**	0	0.003	0	0	0.002	NA	0.001	0.001	0.001	0.002	0.022	0.009	0.007	0.001	0.17	1	0.033	0.001	0.005	0.013	0.005
**rs237900**	0.017	0.034	0.005	0.015	0.003	NA	0.034	0.011	0.041	0.005	0.001	0.026	0.082	0.019	0.195	0.033	1	0.025	0.086	0.001	0.086
**rs61740241**	0.003	0.002	0	0.019	0.001	NA	0.126	0.001	0.004	0.016	0.002	0.001	0.003	0	0.005	0.001	0.025	1	0.002	0.006	0.002
**rs4686302**	0	0.001	0.051	0.075	0.002	NA	0.006	0.004	0.012	0.068	0.003	0.209	0.028	0.002	0.443	0.005	0.086	0.002	1	0.129	0.013
**rs2228485**	0.008	0	0.012	0.002	0.02	NA	0.025	0.01	0.104	0.044	0.016	0.01	0.244	0.004	0.209	0.013	0.001	0.006	0.129	1	0.035
**rs62243375**	0.001	0.048	0.047	0.035	0.002	NA	0.009	0.004	0.012	0.028	0.011	0.004	0.019	0.002	0.03	0.005	0.086	0.002	0.013	0.035	1
